# 21.4 BASELINE CLINICAL AND BIOLOGICAL VARIABLES PREDICTING 1 YEAR OUTCOME OF SUBJECTS AT CLINICAL HIGH RISK OF PSYCHOSIS: INSIGHT FROM SHANGHAI AT RISK FOR PSYCHOSIS (SHARP) PROGRAM

**DOI:** 10.1093/schbul/sby014.088

**Published:** 2018-04-01

**Authors:** TianHong Zhang, HuiJun Li, YingYing Tang, Chunbo Li, Kristen Woodberry, Daniel I Shapiro, Margaret Niznikiewicz, Martha E Shenton, Matcheri S Keshavan, William S Stone, Jijun Wang

**Affiliations:** 1 Shanghai Mental Health Center, Shanghai Jiao Tong University School of Medicine; 2 Florida A & M University; 3 Shanghai Mental Health Center, Shanghai Jiao Tong University School of Medicine, Shanghai Key Laboratory of Psychotic Disorders; 4 Beth Israel Deaconess/ Harvard Medical Center; 5 Harvard Medical School, Veterans Affairs Boston Healthcare System; 6 Psychiatry Neuroimaging Laboratory, Brigham and Women’s Hospital, Harvard Medical School, Veterans Affairs Boston Healthcare System; 7 Massachusetts Mental Health Center Public, Beth Israel Deaconess Medical Center, Harvard Medical School

## Abstract

**Background:**

In 2010, the “ShangHai At Risk for Psychosis (SHARP)” study was launched at the Shanghai Mental Health Center (SMHC), the largest outpatient mental health clinic in China. The Chinese SHARP research was led by Dr. Larry Seidman, who was also the PI of the Harvard site of the NAPLS project. He had implemented methods very similar to those used in NAPLS for the identification of clinical high risk (CHR) individuals in Mainland China in studies jointly funded by the United States National Institute of Mental Health and Chinese funding agencies.

**Methods:**

Dr. Seidman began a collaboration with the SMHC by advising us in carrying out an epidemiological study and then received joint funding for an R21 MH093294 (Fogarty/NIH, “Broadening the Investigation of Psychosis Prodrome to Different Cultural Groups”) designed to implement a variety of clinical, neurocognitive and event related potential (ERP) measures in a preliminary study of CHR. That study, which began in April 2012 and ended in March 2015, aimed to build research capacity at the SMHC. He guide us provided 4 in-person research and clinical skills trainings (2 in SMHC, 2 in Boston), translated a widely used CHR diagnostic instrument (the Structured Interview for Prodromal Symptoms/SIPS), and trained China partners to conduct a preliminary study of 100 CHR individuals. Building upon the R21 project, and the North American Prodrome Longitudinal Studies (NAPLS) model, the same group of researchers led by Dr. Seidman is collaborating on an NIMH R01 101052-01 project (2013 to 2016) to examine biomarkers of CHR with 1 year follow-up. The R21 and R01 collaborations between Harvard Medical School (HMS), MIT, Florida A&M University (FAMU), and SMHC investigators have capitalized on the resources and experiences of the Harvard researchers as members of NAPLS, MIT researchers’ leading role in functional magnetic resonance imaging (fMRI), and FAMU researchers’ expertise in bridging western and Chinese cultures to enhance the existing capacity of Chinese researchers studying the biopsychosocial aspects of CHR. Finally, a stratified cohort of 300 CHR participants was recruited between 2012–2015, and followed up for at least 1 year.

**Results:**

With the hope of Dr. Seidman, the SHARP project is ongoing and getting better, larger and stronger. Of the total 417 CHR participants (previous epidemiological survey [CHR, n = 117], R21 [CHR, n = 100], R01 [CHR, n =200]), 349 completed at least a year of follow-up (until August 30, 2017; the longest follow-up case was six and a half years), in which 83 converted to psychosis, and 68 were lost. Preliminary data showed about 20% CHR converted to a psychotic disorder over the course of follow-up, several clinical factors such as 1) functional decline; 2) selected positive symptoms(unusual thoughts and suspiciousness); 3) selected negative symptoms(social anhedonia, expression of emotion, and ideational richness); biological factors such as the P300 auditory ERP; fMRI: Reduced anti-correlation between the bilateral parietal lobule and left dorsolateral prefrontal cortex; Structural MRI: superior temporal gyrus. et al. are account for increasing the risk of conversion to full psychosis.

**Discussion:**

This is the first, well-implemented, longitudinal study of CHR in a low and middle-income country to comprehensively investigate clinical and biological factors in predicting psychosis conversion and illness progression. Dr. Seidman provide a critical step in the implementation of CHR concept in China, just as an obvious need and urgency for prevention and early intervention for Chinese patients with schizophrenia.

